# GlaI digestion of mouse γ-satellite DNA: study of primary structure and ACGT sites methylation

**DOI:** 10.1186/1471-2164-10-322

**Published:** 2009-07-17

**Authors:** Murat A Abdurashitov, Valery A Chernukhin, Danila A Gonchar, Sergey Kh Degtyarev

**Affiliations:** 1 SibEnzyme, 2/12 Ak. Timakov Str, 630117 Novosibirsk, Russia

## Abstract

**Background:**

Patterns of mouse DNA hydrolysis with restriction enzymes are coincided with calculated diagrams of genomic DNA digestion *in silico*, except presence of additional bright bands, which correspond to monomer and dimer of γ-satellite DNA. Only small portion of mouse γ-satellite DNA sequences are presented in databases. Methyl-directed endonuclease GlaI cleaves mouse DNA and may be useful for a detailed study of primary structure and CG dinucleotides methylation in γ-satellite DNA.

**Results:**

We have constructed a physical map and produced experimental patterns of mouse γ-satellite DNA hydrolysis with unique site-specific methyl-directed endonuclease GlaI and several restriction endonucleases. Fifty two DNA fragments of γ-satellite DNA have been cloned and sequenced. We have not observed any mutations of CG dinucleotide in position 208 of monomeric γ-satellite DNA and confirmed 50% methylation of this CG dinucleoitide. A comparison of consensus sequences of arrayed γ-satellite DNA and small blocks of satellite DNA (140 monomers and less) has shown a higher level of mutations and an absence of conserved CG dinucleotide in last ones. A replacement of CG dinucleotide by CA-dinucleotide in positions 178 and 17 in chromosomes 9 and 3, respectively, has been observed in blocks of monomers.

**Conclusion:**

Arrayed γ-satellite DNA from mouse has at least one conservative CG-dinucleotide. Consensus sequences of this DNA and γ-satellite DNA in small blocks of monomers are differing. The last one displays a higher level of CG dinucleotides mutations and an absence of conservative CG-dinucleotide. Presence of conservative and half-methylated CG-dinucleotide supports an idea of importance of this CG dinucleotide methylation/demethylation in arrayed γ-satellite DNA functioning.

## Background

Site-specific methyl-directed DNA endonuclease GlaI is the most interesting enzyme among new bacterial endonucleases, which cleave recognition sites with 5-methylcytosines and don't cut unmodified DNA [1-4]. GlaI substrates have been studied in details and include methylated DNA sequences 5'-Pu(5mC)GPy-3'/3'-PyG(5mC)Pu-5'and 5'-G(5mC)Pu(5mC)-3'/3'-(5mC)GPyG-5' [5,6].

Previously, we studied a cleavage of total mouse DNA with restriction endonucleases [7]. We compared the experimental patterns of DNA hydrolysis with calculated diagrams of genomic DNA fragments distribution, which had been obtained by computer analysis, and found a good correspondence [7]. We showed that patterns of mouse DNA hydrolysis were formed mainly by products of LINE1 repeats and γ-satellite DNA cleavage. Because of high proportion of γ-satellite DNA in a total mouse DNA preparation the bands, which correspond to products of γ-satellite DNA cleavage, were seen much better in electrophoresis gels [7]. We cut mouse DNA with site-specific methyl-directed DNA endonucleases and observed DNA hydrolysis with GlaI only [7].

In this work we have undertaken a detailed study of mouse DNA cleavage with GlaI and analyzed a primary structure of γ-satellite DNA and methylation status of ACGT sites within it.

## Results

### γ-Satellite DNA digestion with GlaI

Earlier we carried out a hydrolysis of total mouse DNA by restriction endonucleases and site-specific methyl-directed DNA endonucleases with subsequent separation of the reaction products by gel-electrophoresis [7]. A theoretical analysis of mouse genomic DNA digestion at recognition sequences of corresponding restriction endonucleases with subsequent construction of DNA fragments distribution diagrams has been performed and has shown a presence of peaks, which correspond mainly to products of LINE1 repeats cleavage [7]. A comparison of observed experimental results and calculation data has demonstrated a good coincidence, except presence of additional bright bands in the experimental cleavage patterns. These bands correspond to monomer and dimer of γ-satellite DNA 234 bp and 468 bp in length, respectively [7]. A proportion of γ-satellite DNA in a total mouse DNA preparation is about 8% [8] and higher than in total DNA preparations from rat and human [7]. This peculiarity allows us to use a total mouse DNA preparation in the experiments on study of mouse γ-satellite DNA hydrolysis.

**Figure 1:**
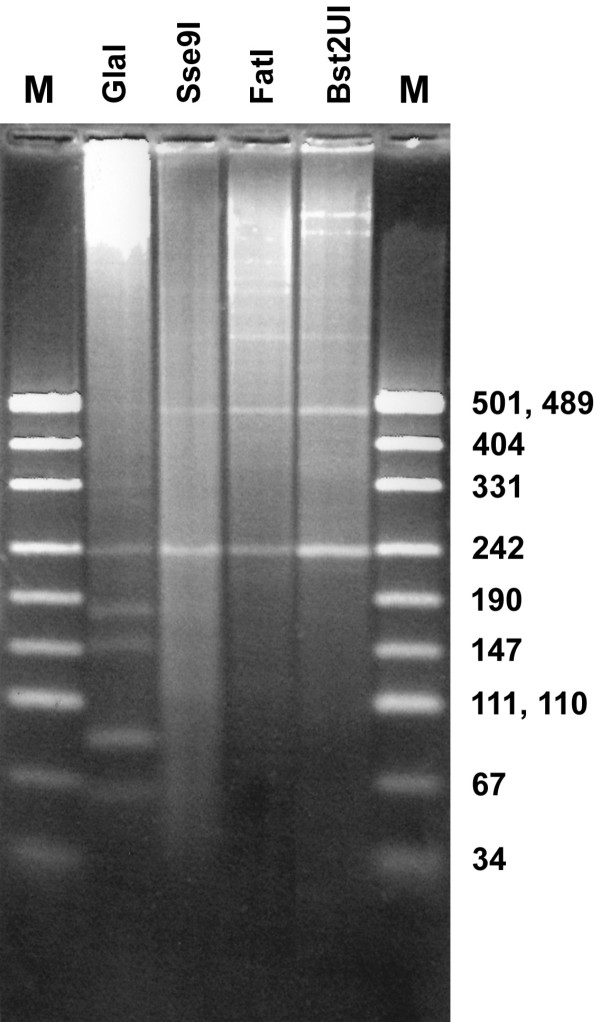
**Mouse genomic DNA cleavage with enzymes GlaI, Sse9I, FatI, Bst2UI**. 1,8% low melting point agarose, Tris-acetate buffer. M – DNA marker pUC19/MspI (the lengths of the fragments are shown at right).

To analyze products of mouse γ-satellite DNA cleavage we have used gel-electrophoresis in PAAG and low melting point agarose. Figure 1 shows patterns of total mouse DNA cleavage with restriction enzymes Sse9I, FatI, Bst2UI and methyl-directed site-specific DNA endonuclease GlaI. In accordance with earlier published data [9,10] DNA hydrolysis with restriction enzymes results in formation of the bands, which correspond to DNA fragments 234 bp and 468 bp and represent monomer and dimer of mouse γ-satellite DNA [11]. GlaI digestion pattern also contains a monomeric γ-satellite DNA fragment. However, unlike other enzyme, GlaI produces a number of shorter fragments, clearly visible in the gel photograph, and this picture is not changed after additional GlaI treatment (data not shown). Comparing to DNA ladder, the lengths of DNA fragments in pattern of GlaI cleavage have been estimated 60, 85, 150, 180 and 234 bp, where the last one is a length of monomeric γ-satellite DNA. In our previous work on mouse DNA hydrolysis with restriction endonucleases we have shown that DNA fragments less than 240 bp are mostly the products of γ-satellite DNA cleavage (except BstX2I and RsaI digestions) [7]. Data on Figure 1 show that an intensity of 234 bp fragment in GlaI pattern is weaker and 468 bp band is missing if compared to other runs. This result corresponds to formation of short fragments in pattern of GlaI digestion from γ-satellite DNA.

### Location of GlaI digestion fragments

**Figure 2:**
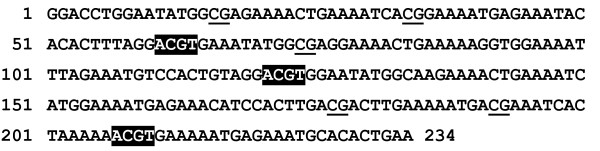
**Consensus sequence of mouse γ-satellite DNA monomer**. CG sites are underlined, ACGT sites (GlaI recognition sites) are shown by inverted color.

To confirm the origin of DNA fragments in pattern of GlaI digestion and to determine their primary structure all five DNA bands have been eluted from gel and cloned into pUC19. DNA insertions of 50 plasmids have been sequenced. Primary structures of 39 insertions correspond to DNA sequence of consensus monomeric γ-satellite DNA [11], which is given in Figure 2.

According to this consensus sequence, the recognition sites of restriction enzymes Bst2UI (CCWGG), FatI (CATG) and Sse9I (AATT) are located in positions 4, 150 and 98 of γ-satellite DNA, respectively. In a course of γ-satellite DNA hydrolysis with these enzymes monomer and multimers are formed (Figure 1). Analysis of consensus DNA sequence (Figure 2) with methylated CG dinucleotides has shown that only three GlaI recognition sites with nucleotide sequence ACGT are present in the main monomeric γ-satellite DNA in positions 61, 121 and 207.

**Figure 3:**
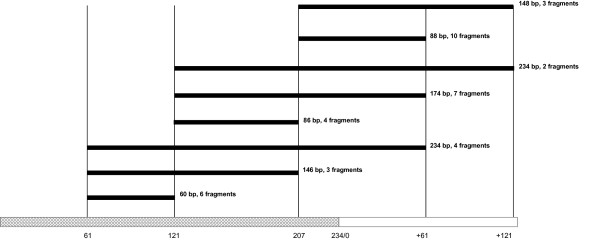
**Correspondence of the sequenced GlaI DNA fragments to the mouse γ-satellite consensus sequence**. Monomeric γ-satellite DNA is shown in grey, sequenced GlaI-fragments – in black. Vertical lines show ACGT sites (positions are indicated at bottom).

In Figure 3 we have presented a diagram with location of all 39 sequenced fragments in monomeric γ-satellite DNA based on their primary structure and positions of GlaI recognition sites. Thus, we have determined DNA sequence of six insertions from lower 60 bp band (positions on γ-satellite DNA 61–121), fourteen insertions of 85 bp band (including four 86 bp fragments 121–207 and ten 88 bp fragments 207-61), six 150 bp insertions (including three 146 bp fragments 61–207 and three 148 bp fragments 207-121), seven 174 bp insertions (121-61) from 180 bp band and six 234 bp fragments. This set includes all possible DNA fragments with a length of 234 bp and less, which are produced in the course of limited GlaI hydrolysis of γ-satellite DNA at positions 61, 121 and 207, except a monomer 207–207. A primary structure of all 39 sequenced fragments and their alignment are presented in Additional file 1 (top 39 sequences of the multiple alignment). Data in Additional file 1 show that in all cases GlaI cleaves only ACGT sites in position 61, 121 and 207. So, we haven't observed any non-specific hydrolysis of γ-satellite DNA with GlaI.

### Incomplete hydrolysis of ACGT sites in γ-satellite DNA

As can be seen from a map in Figure 3, a complete hydrolysis of fully methylated γ-satellite DNA should produce only three DNA fragments: 60 bp, 86 bp and 88 bp. These fragments correspond to the bottom bands 60 bp and 85 bp in Figure 1, taking into account a double intensity of the last one.

However, experimental pattern of GlaI hydrolysis (Figure 1) includes three additional bands 150 bp, 180 bp and 234 bp, which are a result of partial hydrolysis of γ-satellite DNA because of mutations [7,11,12] and undermethylation [11,12] of ACGT sites. A ratio of molar intensities of DNA bands obtained in GlaI cleavage pattern (234 bp, 180 bp, 150 bp, 85 bp and 60 bp) has been determined as 0.2 : 0.3 : 0.3 : 1.5 : 1.

According to sequencing data of the cloned insertions, 174 bp DNA fragment corresponds to 180 bp band, 146 bp and 148 bp DNA fragments are presented in 150 bp band and 234 bp is a monomeric γ-satellite DNA.

In Table 1 we have summarized data on DNA fragments, which contain unmethylated and mutated GlaI recognition sequence(s). There are approximately 24%, 30% and 2% mutated sites in positions 61, 121 and 207, respectively. Additionally, 3%, 7% and 50% of unmethylated sites have been found in the same positions. The last data correspond to earlier published results on 50% methylation of CG dinucleotide in position 208, whereas other CG dinucleotides (underlined on Figure 2) are extensively methylated [11,12]. In total, 27%, 37% and 52% of tetranucleotides at positions 61, 121 and 207, respectively, are resistant against GlaI hydrolysis and corresponding probabilities of these positions cleavage are 73%, 63% and 48%. Based on the probability data we have calculated a ratio of molar intensities of DNA bands obtained in GlaI cleavage (234 bp, 174 bp, 146 bp + 148 bp, 86 bp + 88 bp and 60 bp) as 0.37 : 0.52 : 0.45 : 1.41 : 1. A comparison of these calculations and molar intensities of DNA bands in experiment has shown a similarity of both ratios.

**Table 1:** DNA fragments with mutated or uncleaved ACGT sites

**Fragment**	**Length, bp**	**Positions of fragment in consensus**	**Sequence of non-hydrolysed site at position**		
			
			**121**	**207**	**61**
G150-5N	148	207 – +121	-	-	**ACGT**

G150-1N	147	207 – +121	-	-	ACGA

G150-12N	148	207 – +121	-	-	ATGT

G150-29N	148	207 – +121	-	-	ATGT

G150-33N	148	207 – +121	-	-	ACAT

G234-4	234	121 – +121	-	**ACGT**	TCGT

G234-8N	234	121 – +121	-	**ACGT**	ACTT

G180-1	174	121 – +61	-	**ACGT**	-

G180-3	174	121 – +61	-	**ACGT**	-

G180-6	174	121 – +61	-	**ACGT**	-

G180-2N	174	121 – +61	-	**ACGT**	-

G180-3N	173	121 – +61	-	**ACGT**	-

G180-4N	174	121 – +61	-	**ACGT**	-

G180-6N	173	121 – +61	-	**ACGT**	-

G234-3	234	61 – +61	**ACGT**	**ACGT**	-

G234-13	234	61 – +61	**ACGT**	**ACGT**	-

G234-6d	234	61 – +61	ACAT	**ACGT**	-

G234-9N	234	61 – +61	ACAT	**ACGT**	-

G150-4-2	146	61 – 207	AGGT	-	-

G150-6	146	61 – 207	TCGT	-	-

G150-6N	145	61 – 207	GCAT	-	-

G150-21N	146	61 – 207	ACGA	-	-

G150-22N2	146	61 – 207	AAGT	-	-

G150-24N	146	61 – 207	ACAT	-	-

S234-3	235	98 – +98	ACAT	CCGT	**ACGT**

S234-15N	233	98 – +98	ACTA	**ACGT**	**ACGT**

A234-3N	232	4 – +4	ACAT	**ACGT**	**ACGT**

A234-4b	234	4 – +4	ACAT	**ACGT**	**ACGT**

A234-3	233	4 – +4	ACAT	**ACGT**	ACAT

S234-13N	234	98 – +98	ACAT	**ACGT**	ACAT

S234-1	234	98 – +98	**ACGT**	**ACGT**	ACAT

Number of fragments with non-hydrolyzed ACGT and their percentage			2/29	13/26	1/34
			6,9%	50%	3,3%

Number of mutated ACGT sites			14/47	1/46	9/38
			29,8%	2,2%	23,7%

**Figure 4:**
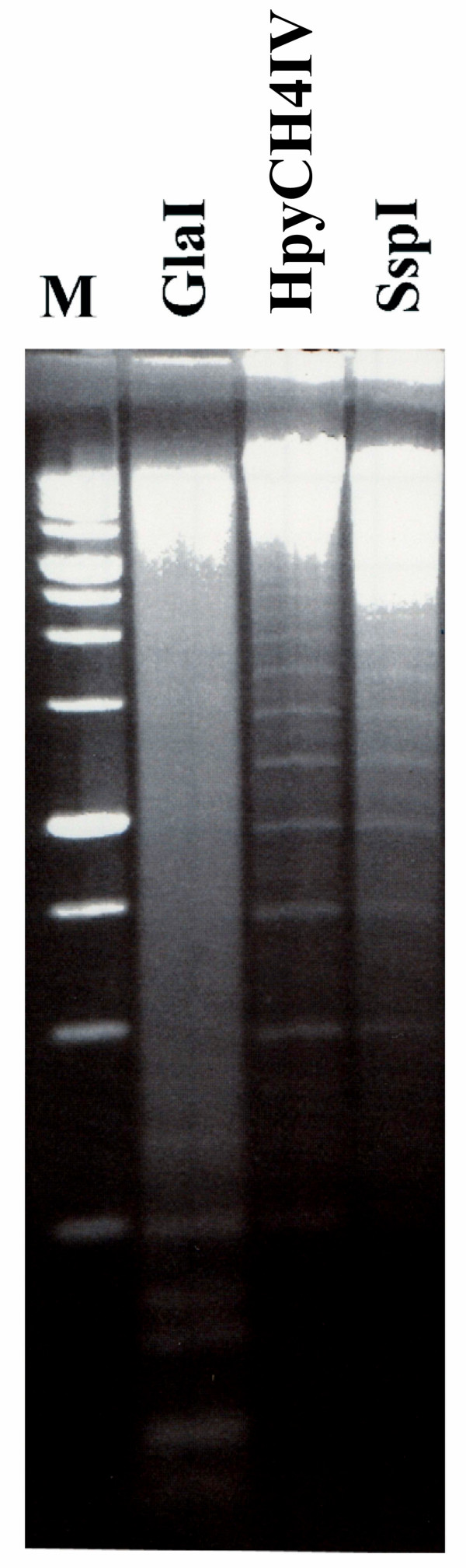
**Mouse genomic DNA cleavage with enzymes GlaI, HpyCH4IV and SspI**. M – DNA marker SE 1 kbp.

Figure 4 demonstrates patterns of total mouse DNA hydrolysis with restriction endonucleases HpyCH4IV, which recognizes unmethylated site ACGT, and SspI (recognition sequence 5'-AATATT-3'). We can see a weak hydrolysis of genomic DNA and similar patterns of incomplete cleavage of γ-satellite DNA with HpyCH4IV and SspI.

### A double digestion of mouse γ-satellite DNA with GlaI and restriction enzymes

**Figure 5:**
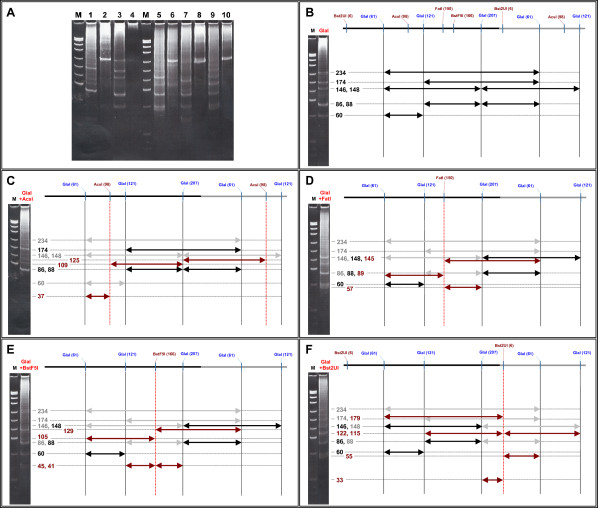
**A physical map of mouse γ-satellite DNA**. A – gel photo of mouse genomic DNA cleavage with restriction enzymes (double digestions). Lanes: 4 – intact DNA; 1, 3, 5, 7, 9 – GlaI added; 1, 2 – AcsI added; 5, 6 – FatI added; 7, 8 – BstF5I added; 9, 10 – Bst2UI added; M – DNA marker pUC19/MspI. B-F – physical maps of mouse γ-satellite DNA with indication of experimentally obtained DNA fragments and theoretically predicted ones. B – GlaI; C – double digestion with GlaI and AcsI; D – double digestion with GlaI and FatI; E – double digestion with GlaI and BstF5I; F – double digestion with GlaI and Bst2UI. Experimental patterns of mouse DNA hydrolysis and DNA ladders are shown on a left side of each figure. Positions of cleavage sites are shown in brackets at the top. Intact GlaI fragments are shown in black, cleaved GlaI fragments are shown in grey, double digestion products are shown in brown. Horizontal dotted lines show a correspondence of predicted fragments (arrows) to those observed on gel photos.

We have carried out the experiments on double digestion of mouse DNA with GlaI and some restriction endonucleases, which have a unique recognition site in γ-satellite DNA. These restriction endonucleases are Bst2UI (recognition site CCWGG at position 4), AcsI (RAATTY, 97), FatI (CATG, 150) and BstF5I (GGATG, 166). The photos of double digestions experiments are provided in Figure 5A. A restriction map of mouse γ-satellite DNA monomer from position 61 to +61 is provided in Figure 5B. This map includes positions of DNA cleavage with GlaI and restriction endonucleases with indication of the obtained fragments and their calculated lengths. As can be seen from Figure 5A, double digestions result in a disappearance of those fragments, which contain restriction enzymes' sites. A treatment of GlaI digestion products with AcsI (Figure 5C) results in hydrolysis of 60 bp, 146 bp and 234 bp DNA fragments. A new fragment 37 bp is a result of 60 bp DNA fragment cleavage. A double digestion with FatI (Figure 5D) destroys all upper fragment of GlaI cleavage pattern but leads to formation of 89 bp and 145 bp DNA fragments. The appearance of 89 bp and 145 bp fragments restores an original picture of 80 bp and 150 bp bands in DNA cleavage pattern and we can see a new 57 bp fragment, which is cut off 86 bp fragment. A similar picture with hydrolysis of all upper fragments of GlaI cleavage pattern is observed with BstF5I double digestion (Figure 5E). A new fragments 129 bp, 105 bp, 45 bp and 41 bp are appeared in digestion products and clearly seen in gel photo. A double digestion of γ-satellite DNA with GlaI and Bst2UI (Figure 5F) results in digestion of 88 bp fragment with formation of two small fragments 33 bp and 55 bp. So, the experimental data on double digestion of γ-satellite DNA with GlaI and restriction endonucleases are coincided with calculated positions of cleavage sites in a physical map.

### Primary structure of γ-satellite DNA

To study a primary structure of γ-satellite DNA in detail, we have cloned additionally 234 bp fragments of AspS9I and Sse9I digestions of mouse γ-satellite DNA [7]. 10 insertions of each digestion have been sequenced. Six Sse9I fragments and seven AspS9I fragments share homology with mouse γ-satellite DNA. These 13 sequences have been aligned with a consensus sequence of monomeric γ-satellite DNA and are presented in Additional file 1, along with primary structures of GlaI hydrolysis fragments. In total, sequencing data of 52 fragments of γ-satellite DNA are provided in Additional file 1. A primary structure of consensus monomeric γ-satellite DNA is given beneath all sequences (see Additional file 1). We haven't observed a mutation of CG dinucleotide in position 208 in all 47 analyzed fragments, whereas we have 7 (from 38 analyzed fragments) mutations of CG dinucleotide in position 62 and 12 (from 47 analyzed fragments) – in position 122.

Earlier, Vissel and Choo cleaved a mouse γ-satellite DNA with MnlI, cloned 30 obtained fragments of monomeric γ-satellite DNA and sequenced insertions of the corresponding plasmids [13]. We have aligned these sequences and provided a consensus primary structure in Additional file 2.

A search for monomeric γ-satellite DNA in the reference mouse genome sequence (NCBI Build 37, [14]) has been made and two small blocks of γ-satellite DNA monomers have been revealed in chromosome 3 and 9. Locations and a primary structure of 30 sequences from the monomers block in chromosome 3 and 140 sequences from the monomers block in chromosome 9 are presented in Additional files 3 and 4, respectively. We have determined consensus sequences of γ-satellite DNA monomers for both blocks and placed them in Additional files 3 and 4 as well. In Table 2 we have summarized data on mutations of CG dinucleotide in all 8 positions in a) 52 fragments of γ-satellite DNA from Additional file 1, b) 30 monomers of γ-satellite DNA from Additional file 2), c) 140 monomers in chromosome 9 from Additional file 3 and d) 40 monomers in chromosome 3 from Additional file 4.

**Table 2:** Mutations of CG-dinucleotide in mouse γ-satellite DNA

**Position**		**16**	**34**	**62**	**74**	**122**	**177**	**192**	**208**
**% of mutated CG dinucleotides**	**DNA fragments (this work)**	10/37	6/37	7/38	6/38	12/47	4/35	7/35	0/46
		**27.0%**	**16.2%**	**18.4%**	**15.8%**	**25.5%**	**11.4%**	**20.0%**	**0%**
	
	**30 MnlI- fragments (Vissel and Choo **[14]**)**	10/30	5/30	3/30	0/30	8/30	1/30	2/30	6/30
		**33.3%**	**16.7%**	**10.0%**	**0%**	**26.7%**	**3.3%**	**6.7%**	**20%**
	
	140 fragments from chromosome 9*	54/140	46/140	63/140	25/140	128/140	108/140	68/140	13/140
		**38.6%**	**32.9%**	**45.0%**	**17.9%**	**91.4%**	**77.1%**	**48.6%**	**9.3%**
	
	40 fragments from chromosome 3**	28/40	14/40	20/40	13/40	38/40	21/40	10/40	16/40
		**70%**	**35%**	**50%**	**32.5%**	**95%**	**52.5%**	**25%**	**40%**

* – region 3000003–3038419 from chromosome 9

** – from chromosome 3 regions:

3000002–3004438

3010698–3015403

3016887–3018046

## Discussion

According to a primary structure of consensus sequence of γ-satellite DNA [11], there are three GlaI recognition sites 5'-A(5mC)GT-3'/3'-TG(5mC)A-3' within monomeric DNA in positions 61, 121 and 207. Patterns of a total DNA hydrolysis with AspS9I and BstSCI have shown that products of γ-satellite DNA digestion have a molar concentration at least 20 times more than a concentration of LINE1 repeats digestions [7]. Because of such difference in DNA concentration we have used a total mouse DNA preparation in study of γ-satellite DNA cleavage with GlaI. The obtained cleavage pattern corresponds to a limited hydrolysis of γ-satellite DNA with GlaI due to mutations of ACGT sites and undermethylation of CG dinucleotides.

Results of double digestion of γ-satellite DNA with GlaI and restriction endonucleases AcsI, Bst2UI, BstF5I and FatI have confirmed positions of enzymes' recognition sites and locations of small (60 bp, 86 bp and 88 bp) and intermediate (146 bp, 148 bp and 174 bp) GlaI digestion fragments in monomeric γ-satellite DNA.

Hydrolysis of γ-satellite DNA with HpyCH4IV and SspI has provided similar patterns of evenly distributed DNA fragments, which are monomers, dimers, trimers, etc. (Figure 4). However, the reasons of such specific digestion of γ-satellite DNA with these restriction enzymes are different. Incomplete hydrolysis of γ-satellite DNA with SspI is explained by mutation of the enzyme recognition sequence [7], whereas HpyCH4IV cleaves γ-satellite DNA due to undermethylation of ACGT site in position 207 (Table 1).

Consensus sequences of γ-satellite DNA fragments obtained in our work (Additional file 1) and published earlier [11] are coinciding. Surprisingly, a consensus sequence, which has been determined for 30 monomeric γ-satellite DNA fragments sequenced in the work of Vissel and Choo (Additional file 2), differs in two positions from a known one [11] (missed A nucleotides in positions 68 and 188).

Vissel and Choo cleaved γ-satellite DNA with MnlI and determined a primary structure of obtained monomers [13]. Earlier Horz and Altenburger [11] studied MnlI digestion of γ-satellite DNA and showed that methylation of CG dinucleotide in position 74 blocks DNA hydrolysis. So, Vissel and Choo isolated only those DNA monomers, which were unmethylated in position 74 in two neighbouring MnlI sites. Besides a distinct consensus sequence, a detailed analysis of these monomers alignment (Additional file 2) has revealed a different distribution of CG dinucleotide mutations in several positions of γ-satellite DNA (see Table 2).

In our study we have observed 18,4%, 25,5% and 0% mutations of CG dinucleotide in positions 62, 122 and 208, respectively (Table 2). Data of Vissel and Choo show that all 30 sequenced fragments don't have mutations in position 74 and have only one mutation of CG dinucleotide in position 177. On the other hand, there are 20% mutations of CG dinucleotide in position 208. An absence of CG dinucleotide mutations in position 74 may be explained, at least partially, by overlapping position 75 of this dinucleotide with MnlI recognition sequence. A discrepancy of CG dinucleotides mutations in position 208 in two works is a consequence of either a very special selection of DNA fragments in a work of Vissel and Choo (see above) or different sources of DNA (a mouse liver in our work and a cell line in the work of Vissel and Choo). Nevertheless, both works demonstrate maximum CG dinucleotides mutations in position 16 (around 30%) and in position 122 (about 26%). Moreover, there is a similar distribution of mutations in these CG dinucleotides: mostly mutated G in position 123 and roughly equal mutated C and G in positions 16 and 17.

In this work we have confirmed data on large proportion of γ-satellite DNA in a total mouse DNA preparation [7]. Vissel and Choo isolated γ-satellite DNA from a total DNA and studied its structure. According to their data γ-satellite DNA is organized into long uninterrupted arrays of between 1,000 monomers and greater than 8,000 monomers, likely, located near chromosome centromers [13].

So, γ-satellite DNA is a prolonged DNA and constitutes a significant part of total DNA. However, we could not find peaks of 234 bp and 468 bp in diagrams of genomic DNA digestion with restriction enzymes, which have one recognition site in γ-satellite DNA monomer [7]. In fact, the most of γ-satellite DNA is not presented in the known databases [7]. Nevertheless, in genomic database there are small blocks of sequenced γ-satellite DNA, which contain 10–140 monomers [14]. DNA sequences of γ-satellite monomers from two such blocks, which are located in chromosomes 3 and 9, have been aligned in this work (Additional files 3 and 4, respectively). Consensus sequences of γ-satellite DNA of these two blocks are almost identical and differ in nucleotides in positions 18 and 179. However, both of them have three identical differences from consensus sequence of arrayed γ-satellite DNA [[11] and this work]: A instead of G in positions 123 and 202, T instead of A in position 233. Besides, CG dinucleotide in position 178 and in position 17 has become CA dinucleotide in consensus sequences of γ-satellite DNA in chromosomes 9 and 3, respectively. A change of G for A in position 123 destroys one of three ACGT sites and consensus sequence of γ-satellite DNA in both blocks of monomers contains only two GlaI recognition sites in positions 61 and 207.

A comparison of Table 2 data between rows 1, 2 and 3, 4 shows a significant difference of CG dinucleotide mutations in γ-satellite DNA from arrays and in small blocks of monomers. We can see more mutation in all 8 positions of CG dinucleotides in the case of blocks of γ-satellite DNA monomers if compare to CG dinucleotides in arrayed γ-satellite DNA. Table 2 shows that in arrayed γ-satellite DNA there is at least one conservative CG dinucleotide, whereas γ-satellite DNA from blocks of monomers doesn't have a conservative CG dinucleotide in any positions.

Absence of mutations [this work] and 50% methylation [[11,12] and this work] of CG dinucleotide in position 208 allow to say about important role of this CG dinucleotide methylation/demethylation in function of arrayed γ-satellite DNA from mouse liver.

## Conclusion

Hydrolysis of total DNA from mouse liver with new site-specific methyl-directed endonuclease GlaI provides a clear digestion pattern, which corresponds to a partial cleavage of γ-satellite DNA at methylated ACGT sites in positions 61, 121 and 207. Determination of primary structure of 39 obtained DNA fragment has confirmed data on extensive modification of CG dinucleoitide in positions 62 and 122 but only 50% modification in position 208. Study of 52 sequenced fragments of γ-satellite DNA has shown that CG dinucleotide in position 208 is highly conservative and its methylation/demethylation is, likely, important for function of γ-satellite DNA arrays.

Consensus sequence of arrayed γ-satellite DNA is coincided with earlier published one and differs from a consensus sequence of γ-satellite DNA in small (140 and less) monomers blocks.

## Methods

### DNA fragments cloning and sequencing

Male A/He mice aged 5–6 months (Breeding Laboratory of Experimental Animals, Institute of Cytology and Genetics, Novosibirsk) have been used in the experiments. Genomic DNA from the animal liver was isolated as described previously [15]. DNA was digested with endonucleases according to manufacturer's recommendations.

DNA fragments, which formed visible bands, were extracted from PAAG as described earlier [16]. The obtained DNA fragments were ligated with pUC19 linearized with SmaI, and *E. coli *XL10-Gold competent cells were transformed with the obtained ligation mixture according to [16]. The obtained plasmids were isolated with NucleoSpin Plasmid Kit (Macherey-Nagel, Germany). The sequences of insertions were determined using ABI Prism 310 Genetic Analyzer ("Applied Biosystems").

### Hydrolysis of chromosomal DNA and electrophresis

The following restriction endonucleases manufactured by SibEnzyme Ltd. were used in the work (the recognition site of respective restriction enzyme is given in the brackets): GlaI (5'-R(5mC)GY-3'), Bst2UI (5'-CCWGG-3'), FatI (5'-CATG-3'), Sse9I (5'-AATT-3'), AcsI (5'-RAATTY-3'), BstF5I (5'-GGATG-3' and 5'-CATCC-3'), SspI (5'-AATATT-3'). HpyCH4IV restriction enzyme (recognition site 5'-ACGT-3') was purchased from New England Biolabs.

Hydrolysis reactions were performed in 40 μl of the reaction mixture containing 6 μg of DNA, SE-buffers recommended by the manufacturer and 3 μl of restriction enzyme at optimal temperatures for 3 h.

Electrophoresis in 8%–10% polyacrylamide gel was used to separate DNA fragments from 40 to 500 bp (6 μg of hydrolyzed DNA was applied on gel in each run). Electrophoresis in 1.8% of low melting point agarose ("Sigma", USA) was used to separate DNA fragments in the range of 200–2000 bp. Electrophoresis in 1% agarose "Type I-A, Low EEO" ("Sigma", USA) was used to separate DNA fragments in the range of 500–20000 bp. 3 μg of hydrolyzed DNA was applied on agarose gel in each run. Tris-acetate buffer was used for electrophoresis in all the cases. After electrophoresis DNA bands were stained with ethidium bromide and photographed in UV light.

The following DNA preparations were used as DNA fragment length markers: 1 kbp DNA ladder and pUC19/MspI DNA ladder (SibEnzyme Ltd., Russia).

### Sequence analysis

BioEdit 7.09 software was used to obtain and refine multiple alignments of all mouse γ-satellite DNA fragments [17]. The blocks of γ-satellite DNA in the sequenced mouse genome were derived from Entrez Genomes View site by searching for "GSAT_MM" keyword [14].

## Authors' contributions

VAC and DAG carried out DNA hydrolysis, cloning and sequencing of DNA fragments. MAA carried out the data analysis and drafted the manuscript. SKD coordinated the project and prepared the final manuscript. All authors have read and approved the final manuscript.

## Supplementary Material

Additional file 1 **Alignment of the sequenced GlaI, Sse9I and AspS9I fragments**. Numbering is given according to consensus sequence [11]. ACGT sites are given with yellow background. Nucleotides which differ from those in consensus are given with blue background. Sse9I fragments (names start with S) and AspS9I fragments (names start with A) are indicated in green. Some pieces of fragments (shown in magenta) were moved in the alignment to fit to a total length of 234 bp. CG dinucleotides are shown in blue. Yellow background indicates ACGT sites (cleaved or not cleaved). Click here for file

Additional file 2 **Alignment of 30 γ-satellite MnlI DNA fragments (Vissell and Choo**, [13]). Numbering is given according to consensus sequence [11]. ACGT sites are given with yellow background. CG dinucleotides are in blue. Consensus sequence obtained from this alignment is given at the bottom (grey background) and positions which differ from those in a known consensus [11] are indicated by blue background. Click here for file

Additional file 3 **Alignment of 140 γ-satellite DNA monomers from chromosome 9**. Explanations are the same as for Additional file 2. Click here for file

Additional file 4 **Alignment of 40 γ-satellite DNA fragments from chromosome 3**. Explanations are the same as for Additional file 2. Click here for file
