# Supplementary material for: GlaI digestion of mouse γ-satellite DNA: study of primary structure and ACGT sites methylation
Source: BMC Genomics. 2009 Jul 17;10:322. doi: 10.1186/1471-2164-10-322 (PMC2722675; doi:10.1186/1471-2164-10-322)
Supplement: Additional file 4 — Alignment of 40 γ-satellite DNA fragments from chromosome 3. Explanations are the same as for Additional file 2. [file 1471-2164-10-322-S4.pdf]

```
                    10        20        30        40        50        60        70        80        90       100       110
          ....|....|....|....|....|....|....|....|....|....|....|....|....|....|....|....|....|....|....|....|....|....|....|...
chr3_1    GGACCTGGAATATGGCGAGAAAACTGAAAATCACGGAAAATGAGAATAACACTCGTTAGGTAGTGAAATATGACGAGGAAAACTAAAAAGGTGGAAAATTTAGAAATGTCCACTGTA
chr3_2    GGACCTGGAATATGGCGAGAAAACTGAAAATCCCGGAGAATGAGAAATACACTCTTTAGGACATGAAATATGGCGATGAAAACTGAAACAGGTGGAAAATATAGAAATGTCCACTGTA
chr3_3    GGACCTGGAATATGGCGAGAAAACTGAAAATCACGGAAAATGAGAAATACACAATTTAGGACGTGAAATATGGCGAGGAAAACTGGAAAAGGTGGAAAATTTAAAAATGTCCACTGTA
chr3_4    GGACCTGGAATATGTCAAGAAAACTGAAAAT-GTGGATCATGAGAAATACACACTTTAGGACCTGAAATATGGCGAGGAAAACTGAAAAAGGTGGAAAATTTAGAAATATCCACTGTA
chr3_5    GGACCTGGAATATGGCGAGAAAACTGAAATTCACGGAAAATGAGAAATACACACTTTAGGACGTGAAATATGGCGAGGAAAACTAAAAA-GGTGGAAAATTTAGAAATATCCACTGTA
chr3_6    GGACCTGGAATTTGGCGAGAAAACTGAAAATCACGAAAAATTAGAAATACACACTTTATGACGTGAAATATGGCGAGGAAAACACAAAAAGTTGGACAATTTACAAATGTCTACTGTA
chr3_7    GGACCTGGAATATGGCGAATAAACTGAAAATCACGGAAAATGAGAAATACACACTTTAGGACGTGAAATATGGCGAGGAAAACTGAAAAAGGTGGAAAATATAGAAATGTGCATTGTA
chr3_8    GGACCTGGAATATGGTGGGAAAAGTGAAAATCACGGAAAATGAGAAATACACACTTTAGGACATGAAATATGGCGAGGAAAACTGAAAAAGGTGAAAAATTTAGAAATATGGACTGTA
chr3_9    GGACCTGGAATATGG-AATATAACTGAAAAT-GTAGATCATGAGAAATAAAAACTTTAGGACGTGAAATATGGCGAGGAAAACTGAAAAATTTGAAAAATTTAGAAATGTGCACTCTA
chr3_10   GGACATGGAATATGGCGAGAAAACTGAAAATCACGAAAAATTAGAAATTCACACTTTATGACGTGAAATATGGCGAGGAAAAGAGAAAAAGACAGAAAATTTAGAAATGTACACTCTA
chr3_11   GGACATGGAATATAGCCAGAAAACTGAAAATCACGGAAAATAAGAAATACACACTTTAGGACGTGAAATATGGCGATGAAAACTTAAAATGTTGGAAAATATAGAAATGTGCACTGTA
chr3_12   GGACATGGAATATAGCCAGAAAACTAAAAATCACGGTAAATAAGAAATACACACTTAAAGACGTGAAATATGGCGAGGAAAACTGAAAAAGTTGGAAAATTCAGAAATGTGCACTGTA
chr3_13   GGACATGGAATATGGTAAGAAAACTGAAAATCATGGAAAATGAGAAACATCCACTTGAGGACTTTTAAAATGACGA-AATCACTAAAAAACGTGAAAAATG-AGAAATGCACACTGTA
chr3_14   GGACCTGGAATATGGCAAGAAAACTGAAAAT-GTGGATCATGAGAAATACACACTTTAGGACATGAAATATGGCGAGGAAAACTGAAAAAGGTGGAAAATTTACAAATGTCCACTGTA
chr3_15   GGACCTGGAATATGGCGAGAAAACTGAAAATCACGGAAAATGAGAAATACACACTATAGGAGGTGAAATATTGCTAGGAAAACTGAAAAAGGTGGAAAATTTAGATATATCCACTGTA
chr3_16   GGACCTGGAATATGGCAAGATAACTGAAAAT-GTGAATCATGAGAAATACACACTGTAGGACTTGAAATATGGCAAGGAAAACTGAAAAAGTTGGAAAATTTAGAAATATCCACTGTA
chr3_17   GGACCAGGAATATGGCAAGGCAACTGAAAATCACGGAAAATGAGAAATACACACTTTAGGACGTGAAATATTGCGAGGAAAACTGAAAAAGGTGAAAAATTTAGAAATATCGACTGTA
chr3_18   GGACCTGGAATATGG-AATATAACTGAAAAT-GTAGATCATGAGAAATAAACACTTTAGTACCTGAAATATGGTGAGGAAAACTGAAAAAGTTGAAAAATTTAGAAATGTGCACTCTA
chr3_19   -GACCTGGAATATGGCAAGAAAACTGAAA-TCACGGAAAATGAGAAATACACACTTTAGGACGTGAAATATGGAGAGGAAAACTGAAAAAGGGGGAAAATTTGGAAATGTCCACTGTA
chr3_20   GGACCTGGAATATGGCGAGAAAACTGAAAATCACGGAAAATGAGAAATACACACTTTAGGACGTGAAATATGGCGAGGAAAACTGAAAAAGGTTGAAAATTTACAAATGTCCACTGTA
chr3_21   GGACCAGGAATATGGCGAGAAAAGTGAAAATCACGGAAAATGA-AAATACACACTTTAAGACGTGAAATATGGCGAGGAAAACTGAAAAAGGTGAAAAATTTAGAAATATCGACTGTA
chr3_22   GAACCTGGAATATGA-AAGATAACTGAAAAT-GTAGATCATGAAAAATAAACACTTTATAACGTGAAATATGGCGAGGAAAACTGAAAAAGGTGGAAAATTTAGAAATATCCACTGTA
chr3_23   GGACCTGGAATATGGAGAGAAAACTGAAAATCACGGAAAATGAGAAATACACACTTTAGGACGTGAAATATGGCAAGGAACACTGAAAAAGGTAGAAAATTTAGAAATATCCACTGTA
chr3_24   GGACCTGGAATATGGCAAGAAAACTGAAAATCACGGAAAATGAGAAATACACACTTTAGGACGTGAAATATGGAGAGGAAAACTGAAAAAGGTGGAAAATTTGGAAATGTCCACTGTA
chr3_25   GGACCTGGAATATGGCAAGAAAACTGAAAATCACGGAAAATGAGAAATACACACTTTAGGACGTGAAATATGGCGAGGAAAACTGAAAAAGGTTGAAAATTTACAAATGTCCACTGAA
chr3_26   GGACCTGGAATATGGCGAAAAAACTGAAAATCACGGAAAATGAGAAATACACACTTTAGGACTTGAAATATGGCAAGGAATACTGAAAAAGGTGGAAAATTTACAAATGTCCACTGTA
chr3_27   GGACCTGGATTTTGGCGAGAAAACTAAAAATCACGGAAAATGAGAAAAACACTAGTTAGGTAGTCAAATATGAGAAGAAATATTGAAAAACGTGAAAAATG-AGAAATGTACACTGTA
chr3_28   GGACATGGAATATGGCAAGAAACCTGAAAATCATGGAAAATGAGAAACATCCACTTGACTACTTAAAAAATGACCAAAATGACTGAAATATGTTAAAAATG-AGAAATGCACACTGTG
chr3_29   GGACATGGAATATGGCAAGAAAACTGAAAATCATGGAAAATGAGAAACATCCACTTTACGACTTGAAAAATGAC-AAAATCACTGAAAAACGTGAAAAATG-AGAAATGCATACTGTA
chr3_30   GGACCTAGAATACGGAAAGATAACTGAAAAT-GTGGATTATGAGAAATACACACTTTAGGACCTGAAATATGGCGAGGAAAACTGAAAAAGGTGGAAAATTTAGAAATGTCCACTGTA
chr3_31   GGACATGGAATATGGCAAGAAAACTGATAATCATGGAAAATGAGAAACATCCACTTGATGACTTGAAAAATGACGA-AATCACTGAAAAACGTGTAAAATG-AGAATTGCACACTGTA
chr3_32   GGACATGGAATATGGCAAGAAAACTGAAAATCATGGAAAATGAGAAATACACACTTTAGGACCTGAAATATGGCGAGGAAAACTGAAAAAGGTGGAAAATTTAGAAATATCCACTGTA
chr3_33   GGACCTGGAAAATGGCGAGAAAACTGAAAATCACGGAAAATGAGAATTACACACTTTAGGACGTGAAATATGGCGAGGAAAACGGAAAAAGGTGGAAAATTTAGAAATATCCACTGTA
chr3_34   GGACCTGGACTATGGCAAGAAAACTGAAAATCACGAAAAATTAGAAATACACACTTTAGGACGTGAAATATGGCGAGGAAAACTGAAAAAGTTGGAAAGTTTAGAAATGTCCACTGTA
chr3_35   GGACCTGGAATATGGAGAGAAAACTGAAAATCACGGAAAATGAGAAATACACACTTTAGGACGTGAAATATGGCGAGGAAAACTGAAAAAGGTGGAAAATTTAGAAATATCCACTGTA
chr3_36   GGACCTGGAATATGGCAAGATAACTGAAAAT-GTGAATCAAGAGAAATACACATTTTAGGACGTGAAATATGGCGAGGAAAACTGAAAAAGGTGGAAAATTTAGAAATATCCACTGTA
chr3_37   GGACCTGGAATATGGCAAGATAACTGAAAAT-GTGAATCATGAGAAATACACACTTTAGGACATGAAATATGGCGAGGAAA-CTGAAAAAGGTGGAAAATTTAGAAATATCCACTGTA
chr3_38   GGACCTGGAATATGGCAAGAAAACTGAAAATCACGGAAAATTAGAAATACACACTTTAGGATTTGAAATATGGCGAGGAAA-TTGAAAAAGGTGGAGAATTTAGAAATGTCCACTGTA
chr3_39   GGACCTGGAATATGGCGAGAAAACTGAAAATCACGGAAAATGAGAAATACACACTTTAGGACATGAAATATGGCGAGGAAAATTGAAAATGGTGGAAAATTTAGAAATGTCCACTGTA
chr3_40   GGACCTGGAATATGGCGAGAAAACTGATAATCACGGAAAATGAGAAATACACACTTTAGGACATGAA-TATGGCAAGAAAAATTGGAAAAGTTGGAAAATTTAGAAATGTCCACTGTA
Consensus GGACCTGGAATATGGCAAGAAAACTGAAAATCACGGAAAATGAGAAATACACACTTTAGGACGTGAAATATGGCGAGGAAAACTGAAAAAGGTGGAAAATTTAGAAATGTCCACTGTA

           120       130       140       150       160       170       180       190       200       210       220       230
          .|....|....|....|....|....|....|....|....|....|....|....|....|....|....|....|....|.--...|....|....|....|....|....|....
chr3_1    GGACATGGAATATGGCAAGAAAACTGAAAATCATGGAAAATGAGAAACATCCACTTGACGAGTTGAAAAATGACGAAATTACT--GAAAAACGTGAAAAAAGAAAAATGCACACTGTA
chr3_2    GGACGTGGAATATGGCAAGAAAACTGAACATCATGGAAAATGAGAAACATCCACTTGACGACTTGAAAAATGACGAAAGTACTTAAAAAACGTGAAAATTGAGAAATGCACACTAGA
chr3_3    GGACTTGGAATAAGGCAAGAAAACTAAAAATCATGGAAAATGAGATACATCCACTTGACGACTTGAAAAATGACGAAATCACT--GAAAAATATGAAAAATGAGAAATGCACACTGTA
chr3_4    GGACATGGAATATGGCAAGAAAACTGAAAATCATGGAAAATGAGAAACATCCACTTGATGACTTGAAAAATGACGAAATCACT--GAAAAACATGTAAAATGAGAATTGCACACTGTA
chr3_5    CGACAAGGATTATGGCAAGAAAACTGAAAATCATGGAAAATGAGAAACATCCAGTTGACGAATTGAAAAATGAGGAAATCAGT--GAAAAACGTGAAAAATGAGAAATGCACAATGTA
chr3_6    GGACATGGAATATGGCAAGAAAACTGAAAATCATGGAAAATGAGAAACATCCACTTGACTACTTGAAAAATGACGAAATCCTT--GAAAAACTTGTAAAATGATAAATGCACACTGTA
chr3_7    GGACCTGGAATATGGCAAGAAATCTGAAAATCATGGAAAATGAGAAACATCCACTTGAGGACTTTTAAAATGACGAAATCACT--AAAAAACGTGAAAAATGAGAAATGCACACTGTA
chr3_8    GGACATGGAATATGGCAAGAAAACTGAAAATCATGGAAAATGAGAAACATCCACTTGACGACTTGAAAAAT-ACGAAATCACT--GAAAAACGTGAAAAATGAGAATTGCACACTGTA
chr3_9    GGACATAGAATATTTCAAGAAAACTGAAAATCATGGAAAATGAGAAACATCCACTTGACGACTTGAAAAATGACGAAATCACT--GAAAAACGTGTAAAATGAGAAATGCACACTGTA
chr3_10   GGACATAGAATATTTCAAGAAAACTGAAAATCATGGAAAATGAGAAACATCCACTTGACGATTTGAAAAATGACGAAATCACT--GAATAAAGTGAAAACTGGGAAATGCATACTGTA
chr3_11   GGACAAGGAATATGGCAAGAAAACTGAAAATCATGGAAAATGAGAAATATCCAATTGACGACTTGAAAAATGACGAAATCACT--GAAAAAAGTGAAAACTGGGAAATGCATACTGTA
chr3_12   GGACCTGGAATATGGCAAGAAAACTAAAAATCACGGAAAATGAGAAATACACACTTTAGGACATGAAATATGGCGAGGAAAACTGATAAAGGTGGAAAATTTAGAAATGTGCACTGTA
chr3_13   GGGCCTGGAATATGGCGAGAAAACTGAAAATCACGGAAAATGAGAAATACACACTTTAGGACATGAAATATGGCGAGGAAAACTGAAAAAGGTGGAAAATTTATAAATGTCCACTGTA
chr3_14   GGACATGGAATATGGCAAGAAAACTGAAAATCATGGAAAATGAGAAACATCCACTTGACTACTTGAAAAATGACGAAATCATT--TAAAAACTTGTAAAATGATAAATGCACACTGTA
chr3_15   GGAAATGGAATATGGCAAAAAAACTGAAAATCATGGAAAATGAGAAACATCCACTTGACGACTTGTAAAAT-ACGAAATCACT--GAAAAACGTGTAAAATGAAAAATACAAACTGTA
chr3_16   GGACATGGAATATGGCAAGAAAACTGAAAATCATGGAAAATGAGAAACATCCACTTGATGACTTGAAAAATGACTAAATCACT--GAAAAACGTGTAAAATGAGAATTGCACACTGTA
chr3_17   GGACATGGAATATGGCAACAAAACTGAAAATAATGGAAAATGAGAAACATCCACTTGATGACTTGAAAAAT-ACCAAATCACT--GAAAAACGTGAAAAATGAGAATTGCACACTGTA
chr3_18   GGCCATAGAATATTTCAAGAAAACTGAATATCATGGAAAATGAGAAACATCCACTTGACGACTTGAAAAATGACAAAATCACT--GAAAAATGTGAAAAAT-AGAATTGCACACTGTA
chr3_19   GGACATGGAATATGGCAAGAAAACTGAAAATCATGGAAAATGAGAAACATCCACGTGACGACTTGAAAAATGATGATATCACT--GAAAAACGTGAAAAATGAGAAATGAATACTGTA
chr3_20   GGACATGGAATATGGCAAGAAAACTGAAAATCATGGAAAATGAGAAACATCCACTTGATGACTTGAAAAATGACGAAATCACT--GAAAAACGTGTAAAATGAGAATTGTACACTGTA
chr3_21   GGACATGAAATATGGCAAGAAAACTGAAAATCATGGAAAATGAGAAAGATCCACTTGACGACTTGAAAAAT-ACGAAATCACT--GAAAAACGTGAAAAATGAGAATTGCACACTGTA
chr3_22   GGACATGGATTATGGCAAGAAAACTGAAAATCATGGAAAATGAGAAACATCCACTTGACGACTTGAAAAATGACGAAATCACT--GAAAAACGTGTAAAATGAGAATTGCACACTGTA
chr3_23   GGACATGGAATATGGCAAGAAAACTGAAAATCATGGAAAATGAGAAACATCCACTTGACGACTTGAAAAATGACGAAATCACT--GAAAAACGTGTAAAATTAGAATTGCACACTGTA
chr3_24   GGACATGGAATATGGCAAGAAAACTGAAAATCATGGAAAATGAGAAACATCCACGTGACGACTTGAAAAATGACGAAATCACT--GAAAAACGTGAAAAATGAGAAATGCATACTGTA
chr3_25   GGACATGGAATATGGTAAGAATACTGAAAATCATGGAAAATGAGAAACATCCACTTTACGACTTGAAAAATGACGAAATCACT--GAAAAACGTGAAAAATGAGAAATGCACACTGTA
chr3_26   GGACATGGAATATGGCAAGTAAACTGAATATCATGGAAAATGAGAAACATCCACTTGAGTATTTGAAAAATGTTGAAATCACT--GAAAAACGTGAAAAATGAGAAATGCACACTGTA
chr3_27   GGACCTGGAATATGGCAAGATAACTGAAAAT-GTGGATCATGAGACATACACAATTTAGGACGTGATATATGGC-AGGGAAATTGAAAAAG-TGGAAAATTTAGAAATGTCCACTGTA
chr3_28   GGACCTGGAATATGGCGAGAAAACTGAAAATCACGGAAAATGAGAAATACACACTTTAGGAAGTGAAATATGGCGAGGAAAACTGAAAAAGGTGGAAAATTTACAAATGTCCACTTTA
chr3_29   GGACATGGAATATGGCAAGTAAACTGAAAATCACGGAAAATGAGAATAATACTCCTTAGGTAGTGAAATATGACAAGAAATATTGAAAAAAAAGAATAA--TGAAAATGCACACTGTA
chr3_30   GGACCTGGACTATGGCAAGAAAACTGAAAATCACGAAAAATTAGAAATACACACTTTAGGACATGAAATATTGTGAGGAAAACTGAAAAAGTTGGAAAGTTTAGAAATGTTCACTGTA
chr3_31   GGACCTGGAATATGGAGAGAAAACTGAAAATCACGGAAAATGAGAAATACACACTTTAGGACGTGAAATATGGCGAGGAAAACTGAAAAAGGTGGAAAATTTAGAAATATCCACTGTA
chr3_32   GGACATGGAATATGGCAAGAAAACTGAAAATCATGGAAAATGAGAAACATCCACTTGATGACTTGAAAAATGACGAAATCACT--GAAAAACGTGTAAAATGAGAAATGCACATTGTA
chr3_33   GGACATGGAATAGGGCAAGAAAACTGAAAATCTTGGAAAATGAGAAATACACACTTTTGGACCTGAAATATGGCGAGGAAAACTGAAAAAGGTGGAAAATTTTGAAATATCCAATGTA
chr3_34   GGACATGGAATATGGCAAGAAAACTGAAAATCATGGAAAATGAGAAACATCCACTTGATGACTTGAAAAATGACGAAATCACT--GAAAAACGTGTAAAATGAGAATTGCACACTGTA
chr3_35   GGACATGGAATATGGCAAGAAAACTGAAAATCATGGAAAATGAGAAATATCCACTTGACGACTTGAAAAATGACGAAATAACT--GAAAAACGTGAAAAATGAGAAATACACACTGTA
chr3_36   GGACATGGAATATGGCAAGAAAACTGAAAATCATGGAAAATGAGAAACATCCACTTGACGACTTGAAAAAT-ACGAAATCACT--GAAAAACGTGAAAAATGAGTATTGCACACTATA
chr3_37   GGAAATGGAATATGGCAAGAAAACTGAAAATCATGCAAAATGAGAAACATCCACCTGAGGACTTGAAAAATGACGAAATAACT--AAAAAATGAGAAAAATGAGAAATGCACACTGTA
chr3_38   GGACGTGGATTATGGCAAGAAAACTTAAAATCATGGAAAATGAGAAACATCCACTTGACTACTTGAAAAATGACAAAATCACT--GAAAAACGTGAAAAATGAGAAATGCACACTGTA
chr3_39   GGACATGGAATATGGCAAGAAAACTGAAAATCATGGAAAATGAGAAACATCCACTTGACGACTTGAAAAATGACGAAATCACT--CAAAAACGTGAAAAATGAGAAATGCACACTCTA
chr3_40   GGACATGGAATACGGCGAGAAAACTGAAAATCATGGAAAATGACAAACATCCACTTGAATACTTGAAAAATGACGAAATCACT--GAAAAACGTGAAAAATGAGAAATGCACACTGTA
Consensus GGACATGGAATATGGCAAGAAAACTGAAAATCATGGAAAATGAGAAACATCCACTTGACGACTTGAAAAATGACGAAATCACT--GAAAAACGTGAAAAATGAGAAATGCACACTGTA
```
