# Supplementary material for: GlaI digestion of mouse γ-satellite DNA: study of primary structure and ACGT sites methylation
Source: BMC Genomics. 2009 Jul 17;10:322. doi: 10.1186/1471-2164-10-322 (PMC2722675; doi:10.1186/1471-2164-10-322)
Supplement: Additional file 2 — Alignment of 30 γ-satellite MnlI DNA fragments (Vissell and Choo, [13]). Numbering is given according to consensus sequence [11]. ACGT sites are given with yellow background. CG dinucleotides are in blue. Consensus sequence obtained from this alignment is given at the bottom (grey background) and positions which differ from those in a known consensus [11] are indicated by blue background. [file 1471-2164-10-322-S2.pdf]

```
          70        80        90       100       110       120       130       140       150       160       170       180
          -.|....|....|....|....|....|....-|....|....|....|....|....-|....|....|....|....|...-.|....|...-.|....|....|....|....|....
2C        -AATGGCGAGGAAACCTGAAAAAGGTGGAAAA-TTTTGAAATGTCCGCTGAAGGAC-T-AGAATATGGCAAGAAAACTGAAAA-TCATGGAAAA-TGATAAACATCCACTTGACGACT-GA
8E        -TATGGCGAGGAAAGCTGAAAAAGGTGGAAAA-TTTAGAAATGTCCACTGTAGGACATTGGAATATGGCAAGAAAACTGAAAA-TCATGGAAAA-TGAGAAACATCCACTTGACCACTTGA
10A/2     -TAAGGCGAGGAAAACAGAAAAAGTTGGATAA-TTTGGAAATGTCCACTCTAGCACAT-GGAATACGGCAGGAAA-CTGAAAA-TCATGGAAAA-TGAGAAACATCCACTTGACGACTTGA
11B       -TAGGGCGAGGAAAACTGAAAAAGGTGGAAAA-TGTAGAAATGTCCACTGTAGGACAT-GGAATATGGCAAGAAAACTGAAAA-TCATGGAAAAATGAGAAACATCCACTTGACGACTTGT
11D       -TATGGCGAGGAAAACTGAAAAAGGTGGAAAA-TTTAGAAATGTCCACTGTAGGACAT-GGAATATGGCAAGAAAACTGAAAA-TCATGGAAAA--AAGAAACATCCACTTGACGAGTTGA
9F/2      -TATGGCGAGGAAAAATGAAAAAGGTGGAAAA-TTTAGAAATGTCCACTGTAGGACAT-GGAATATGGCAAGACAACTGAAAA-TCATAAAGAA-TGAGAAACATCCACTTGACGACTTGA
10B       -TATGGCGAGGAAAACTGAAAAAGGTGGAAAA-TTTAGAAATGTCCACTGTAGGACGT-GGAATATGGCAAGAAAACTGAAAA-TCATGGAAAA-TGAGAAACATCCACTTGACGACTTGA
6A        -TATGGCGAGGATAACTGAAAAAGGTGGAAAAATTTAGAAATGTCCACTGTAGGACGT-GGAATATGGCAAGAAAACTGAAAA-TCATGGAAAA-TGAGAAACATCCACTTGACGACTTGA
10G       -TATGGCGAGGAAAACTGAAAAAGGTGGAAAA-TTTTGAAATGTCCACTGTAGGACGT-GGAATATGGCAAGAAAACTGAAAA-TGATGGCAAA-TGAGAAACATCCACTTGTCGACTTGA
4B        -TATGGCGAGGAAAACTGAAAAAGGTGGAAAA-TTTAGAAATGTCCACTGTAGGACGT-GGAATATGGCAAGAAAACTGAAAA-TCATGGAAAA-TGAGAAACATCCACTTGTCGACTTGA
10I       -TATGGCGAGGAAAAATGAAAAATTTGGAAAA-TTAAGAAATGTCCACTGTAGGACGT-GGAATATGGCAAGAAAACTGAAAA-TCATGGAAAA-TGAGAAACATCCACTTGACGACTTGA
8I6       -TATGGCGAGGAAAGCTGAAAAAGGTGGAAAA-TTTAGAAATGTACACTGTAGGACGT-GGAATATGGCAAGAAAACTGAAAA-TCCTGGAAAA-TGAGAAACATCCACTTGACGACTTGA
7A        -TATGGCGAGGAAAACTGAAAAAGGTGGAAAA-TTTAGAAATGTCCACTGTAGGACGT-GGAATATGGCAAGAAAACTGAAAA-TCATGGAAAA-TGAGAAACATCCACTTGACGACTTGA
9F/1      -TAAGGCGAGGAAAACTGAAAAAGGTGGAAAA-TTTAGAAATGTCCACTGTAGGACGT-GGAATATGGCAAGAAAACTGAAAA-TCATGGAAAA-TGATAAACATCCACTTGACGACTTGA
10H/2     -TATGGCGAGGAAAACTG-AAAAGGTGGAATA-TTTAGAAATGTCCACTGTAGGACAT-GAAATATGGCAAGAAAACTGAAAA-TCATGGAAAA-TGAGAAACATCCACTTGACGACTTGA
1A        -TATGGCGAGGAAAAATC-AAAAGGTGGAATA-TTTAGAAATGTCCACTGTAGGACTT-GAAACATGGCAAGAAAACTGAAAA-TCATGGAAAA-TGAGAAACATGCCCTTGACGACTTGA
10C       -TATGGCGAGGAAAACAAAAAAAGGTGGAATA-TTTAGAAATGTCCACTGTAGGACGT-GCAATATGGCAAGAAAACTGAAAA-TCATGGAAAA-TGAGAAACATCCACTTGACGACTTGA
4E8       -TATGGCGAGGAAAACTGAAAAAGGTGGAATA-TTTAGAAATGTCCACTGTAGGACGT-GGAATATGGCAAGAAAACTGAAAAATCATGGAAAA-TGAGAAAAATCCACTTGACGACTTGA
3A        -TATGACGAGGAAAACTGAAAAAGGTGGAAAA-TTTAGAAATGTCCACTTTAGGACGG-TGAATATGGCAAGAAAACTGAAAA-TCATGGAAAA-TGAGAAACATCCAGTTGACGACTTCA
8H        ATATG-CGAGGAAAACTGAAAAAGGTGGAAA--TTTAGAAATGTCCACTGTAGGACGT-GGAATATGGCAAGAAAACTGAAAA-TCATGGAAAA-TGAGAAACATCCACTTGACGACTATA
4D        -TATGACGAGGAAAACTGAAAAACGTTGGAAAATTTAGAAATGTCCACTGTAGGACGT-GGAATCTGGCAAGAAAACTGAAAA-TCATGGAAAA-TGAGAAAGATGGAGTTGCCGACTTGA
4A        -TATGGCGAGGAAAACTGAAAAAGTTGGAAAA-TTTAGAAATGTCCACTTTAGGACGT-GGAATATGGCAAGAAACGTGAAAA-TCATGGAAAA-TGAGAAACATCCACTTGACGACTTGA
8J        -TATGGCGAGGAAAGCTGAAAAAGTTGGAAAA-TTTAGAAATGTCCACTCTAGGACGT-CGAATATGGCAAGAAAACTGAAAA-TCATGGAAAA-TGAGAAACATCCACTTGACGACTTTA
10E       -TATGGCGAGGAAAACTGAAAAAGGTGGAAAAATTTAGAAATGTCCACTGTAGGACGT-GGAATATGGCAGGAAAAATGAAAA-TCATGGAAAA-TGAGAAATATCTACTTGACGACTTGC
11G       -TATGGCGAGGAAAACTGAAAAAGTTGGAAAA-TTTAGAAATGTCCACTGTAGGACGT-GGAATATGGCAAGAAAACTGAAAA-TCATGGAAAA-TGAGAAACATCCACTTGACGACTTGA
5A        -TATGTCGAGGAAAACTGAAAAAGGTGGAAAA-TTTAGAAATGTCCACTGTAGGACGT-GGAATTTGGCAAGAAAACTTAAAA-TCATGGAAAA-TGAGAAACATCCACTTGACGACTTGA
8G        -TATGTCGAGGAAAACTGAAAAA-GTGGAAAA-TTTAGAAATGTCCACTGTAGGACGT-GGAATTTGGCAAGAAAACTTAAAA-TCATGGAAAA-TGAGAAACATCCACTTGACGACTTGA
8A        -TATGGCGAGGAAAACTGAAAAAGGTGGAAAA-TTTAGAAATGTCCACTGTAGGACGT-GGAAAATGGCAAGAAAACTGAAAA-TCATGGAAAA-TGAGAAACATCCACTTGTCGACTTAA
9D        -TATGGCGAGGAAAACTGAAAAAGGTGGAAAA-TTTAGAAATGTCCACTGTAGGACTT-GGAATATGGCAAGAAAACTGAAAT-TCATGGAAAA-TGAGAAACATCCACTTGACGACTTGA
7B        ---------------CTGAAAGAGGTGGAAAA-TTTAGAAATGTTCACTCTAGGACGC--GAATATGGCAAGAAAACTAAAAA-TCATGGAAAA-TGCGAAACATCCACTTGACGACTTGA
Consensus -TATGGCGAGGAAAACTGAAAAAGGTGGAAAA-TTTAGAAATGTCCACTGTAGGACGT-GGAATATGGCAAGAAAACTGAAAA-TCATGGAAAA-TGAGAAACATCCACTTGACGACTTGA

              190       200       210       220       230  1      10        20        30        40        50        60
          |....|....|-....|....|.--...|....|....|....|....|....|...|....|....|....|....|....|....|....|....|....|....|....|...
2C        AAAATGACGAAA-TCACTGAAAA--ACATGAAAAATGAGAAATGCACACTGTAGGATCTGGAATATGGCGAGAAAACTGAAAATCACGTAAAATGAAAAATACACACTTTAGGACGTGAA-
8E        AAA-TGACGAAA-TCACTGAATA--ACTTGAAAAATGAGAAATGCACACTGTAGGACCTGGAATATGGCGAGAAAACTGAAAATGACGGAAAATGATAAATACACACTTTAGGACGTGAA-
10A/2     AAA-TGACGAAA-TCACTGAAAA--ACGTGAAAAATGAGAAATGCACACTGTAGGACCTGGAATATGGCGAGAAAACTGAAAATCA-GGAAGATGAGAAATACACACTTTAGGACGTGAAA
11B       AAA-TGACGAAA-TCACTGAAAA--ACGTGAAAAATGAGAAATGCACACTGTAGGGCCTGAAATATGGCAAGAAAACTGAAAATCACGGAAAATGAGAAATACACACTTTAGTACGTGAA-
11D       AAA-TGACGAAA-TCACTAAAAA--ACGTGAAAAATGAGAAATGCACACTGAAGGACCTGGAATATGGCGAGAAAACTGAAAATCACGGAAAAGGAGAAATACACACTTTAGGACGTGAA-
9F/2      AAA-TGACGAAA-TCACAAACAT--ACGTGAAAAATGAGAAATGCACACTGAAGGACCTGGAATATGGCGAGAAAACTGAAAATCACGGGAAATGAGATATACACACTTTAGGACGTGAA-
10B       AAAATGACGAAA-TCACTAAAAA--ACGTGAAAAATGAGAAATGCACACTGAAGGACCTGGAATATGGCGAGAAAACTGAAAATCACGGAAAATGAGAAATACACACTTTAGGACGTGAAA
6A        AAA-TGACGAAA-TCACTAAAAA--ACGTGAAAAATGAGAAATGCACACTGAAGGACCTGGAATATGGCGAGAAAACTGAAAATCACGGAAAATGAGAAATACACACTTTAGGACGTGAA-
10G       AAA-TGACGAAA-TCACCAAAAT--ACGTGAAAAAAGTGAAATGCCCACTGAAGGACCTGGAATATGGCGAGAAAACTGAAAATCATGGAAAATGAGAAATACACACTTTAGGACGTGAA-
4B        AAA-TGACGAAA-TCTCTAAAAA--ACGTGAAAAATGAGAAATGCCCACTGAAGGACCTGGAATATGGCAAGAAAACTGAAAATCACGGAAAATGAGAAATACACACTTTAGGACGTGAA-
10I       AAA-TGACGAAA-TCACTAAAAA--ACGTGAAAAATGAGAAATGCCCACTGAAGGACCTGGAATATGGCGAGAAAACTGAAAATCACGGAAAATGAGAAATACACACTTTAGGACGTGAA-
8I6       AAA-TGACGAAA-TCACTAAAAA--ACGTGAAAAATGAGAAATGCCCACTGAAGGACCTGGAATATGGCGAGAAAACTAAAAATCACGGAAAATGAGAAATACACACTTTAGGACGTGAA-
7A        AAA-TGACGAAA-TCACTAAAAT--TCGTGAAAAATGAGAAATGCACACTGAAGGACCTGGAATA-GGCAAGAAAACTGAAAATCACGGAAAATGAGAAATACACACTTTAGGACGTGAA-
9F/1      AAA-TGACGAAA-TCACTAAAAT--ACGTGAAAAATGAGAAATGCACACTGAAGGACCTGTAATATGGCGAGAAAACTGAAAATCACGGAAAATCACAAATACACAATTTAGGACGTGGTA
10H/2     AAA-TGACGAAA-TCACTAAAAA--ACGTGAAAAATGAGAAATGCACACTGAAGGACCTGGAATATGGCGAGAAAACTGAAAATCACGGAAAATGAGAAATACACACTTTAGGTCGTGAA-
1A        AAA-TGACAAAA-TCACTAAAAA--ACGTGAAAAATGAGAAATGCACACTGAAGGACCTGGAATATGGCGAGAAAACTGAAAATCACGGAAAATGAGAAATACACACTTTAGGACATGAA-
10C       AAA-TGACGAAA-TCACTAAAAA--ACGTGAAAAATGCGAAATGCTCACTGAAGGACCTGGAATATGGCTAGAAAACTGAAAATCACAGAAAATGAGAAATACACACTTTAGGACGTGAA-
4E8       AAA-TGACGAAAATCACTAAAAT--ACGTGAAAAATGAGAAATGCACACTGTAGGACCTGGAATATGGCGAGAAAACTGAAAATCACGGAAAATGAGAAATACACACTTTAGGACGTGAA-
3A        AAA-TGACGAAA-TCACTGAAAAA-ACGTGAAAAATGAGAAATGCACACTGAAGGACCAGGAATATGGCGAGAAAACTGAAAATCACGGAAAATGAGAAATACACACTTTAGGACGTGAA-
8H        AAA-TGACGAAA-TCACTAAAAA--CCGTCAAAAATGAGAAATGCACACTGAAGGACCTGGAATATGGCGAGAAAACTGAAAATCACGGAAAATGAGAAATACACACTTTAGGACATGAA-
4D        AAA-TGACGAAA-TCACTAAAAA--ACGTGAAAAATGAGAAATGCACACTGAAGGACCTGGAATATGGCGAGAAAACTGAAAATTACAGAAAATGAGAAATACA-AATTTAGGACGTGAA-
4A        AAA-TGACGAAA-TCACTAAAAA--ACGTGAAAAATGAGAAATGCACTCTGAAGGACCTGGATTATGGCGAGAAAACTGAAAATCACGCAAAATGAGAAATACACACTTTAGGACGTGAA-
8J        AAA-TGACGAAA-TCACTAAAAT--ACGTGAAAAATGAGAAATGCACACTGAAGGACCTGGAATATGGAGAGAAAACTGAAAATCACGGAAAATGAGAAATACACACTTTAGGACATGAA-
10E       AAA-TGACAAAA-TCACTGAAAA--AAGTGAAAAA--AGAAATGCACACTGTAGGACCTGGAATATGGTGAGAAACCTGAAAATCACGGAAAATGAGAAATACACACTTTAGGACGTGAA-
11G       AAA-TGACGAAA-TCACTGAAAAAACTGTGAAAAATGAGAAATGCACACTGTTGGACCTGGAATATGGCGAGAAAACTGAAAATTACGGAAAATGTGAAATACACACTTTAGGACGTGAA-
5A        AAAATGACGAAA-TCACTGAAAA--ACATGAAAAATGAGAAATGCACACTGTAGGACATGGAATATGGCAAGAAAACTGAAAATTACGGAAAATGAGAAATACCTATTTTAGGTCGTGAA-
8G        AAA-TGACGAAA-TCACTGAAAA--ACATGAAAAATGAGAAATGCACACTGTAGGAACTGGAATATGGCAAGAAAACTGAAAATCACGGAAAATGAGAAATACACACTTTAGGTCGTGAA-
8A        AAA-T-ACGGAA-TCACTGAAAA--ACGTGAAAAATGAGAAATGCACACTGTAGGACCTGGAATATGGGGAGAAAACTGAAAATTACGGAAAATGAGAAATACCTATTTTAGAACGAGAA-
9D        AAA-TAACGAAA-TCACTGAAAA--ACGT-AAAAATGAGAAAAGCACACTGTAGGACATGGAATATGGAGAGAAAACAGAAAATCACGGAAAATGAGAAATACACACTTTAGGACGTGAA-
7B        AAA-TGACGAAA-TCACTGGAAA--ACGTGAAAAATGAGAAATGCACACTGTAGGACCTGGAATATGGCGAGAAAACTGAAAATCAAGTAAAATAAGAAATATACACTTTGGGACGTGAA-
Consensus AAA-TGACGAAA-TCACTAAAAA--ACGTGAAAAATGAGAAATGCACACTGAAGGACCTGGAATATGGCGAGAAAACTGAAAATCACGGAAAATGAGAAATACACACTTTAGGACGTGAA-
```
